# Proton therapy (PT) combined with concurrent chemotherapy for locally advanced non-small cell lung cancer with negative driver genes

**DOI:** 10.1186/s13014-023-02372-8

**Published:** 2023-11-16

**Authors:** Yonglong Jin, Shosei Shimizu, Yinuo Li, Yuan Yao, Xiguang Liu, Hongzong Si, Hideyuki Sakurai, Wenjing Xiao

**Affiliations:** 1 Department of Radiotherapy, The Affiliated Hospital of Qingdao University, Qingdao, China; 2 School of Public Health, Qingdao University, Qingdao, China; 3 Department of Radiation Oncology, University of Tsukuba Hospital, Tsukuba, Japan; 4 Department of Radiotherapy, YIZHOU Cancer Hospital, Qingdao, China; 5 Graduate School of Environmental Science, Hokkaido University, Sapporo, Hokkaido Japan

**Keywords:** Non-small cell Lung cancer(NSCLC), Negative driver genes, Proton therapy, Cardio-pulmonary function, DVH

## Abstract

**Purpose:**

To discuss the optimal treatment modality for inoperable locally advanced Non-Small Cell Lung Cancer patients with poor physical status, impaired cardio-pulmonary function, and negative driver genes, and provide clinical evidence.

**Materials and methods:**

Retrospective analysis of 62 cases of locally advanced non-small cell lung cancer patients with negative driver genes treated at Tsukuba University Hospital(Japan) and Qingdao University Affiliated Hospital(China).The former received proton therapy with concurrent chemotherapy, referred to as the proton group, with 25 cases included; while the latter underwent X-ray therapy with concurrent chemoradiotherapy followed by 1 year of sequential immunomodulatory maintenance therapy, referred to as the X-ray group, with 37 cases included.The treatment response and adverse reactions were assessed using RECIST v1.1 criteria and CTCAE v3.0, and radiotherapy planning and evaluation of organs at risk were performed using the CB-CHOP method.All data were subjected to statistical analysis using GraphPad Prism v9.0, with a T-test using *P* < 0.05 considered statistically significant.

**Results:**

(1)Target dose distribution: compared to the X-ray group, the proton group exhibited smaller CTV and field sizes, with a more pronounced bragg peak.(2)Organs at risk dose: When comparing the proton group to the X-ray group, lung doses (V5, V20, MLD) and heart doses (V40, Dmax) were lower, with statistical significance (*P* < 0.05), while spinal cord and esophagus doses showed no significant differences between the two groups (*P* > 0.05).(3)Treatment-related toxicities: The incidence of grade 3 or higher adverse events in the proton group and X-ray group was 28.6% and 4.2%, respectively, with a statistically significant difference (*P* < 0.05). In terms of the types of adverse events, the proton group primarily experienced esophagitis and pneumonia, while the X-ray group primarily experienced pneumonia, esophagitis, and myocarditis. Both groups did not experience radiation myelitis or esophagotracheal fistula.(4)Efficacy evaluation: The RR in the proton group and X-ray group was 68.1% and 70.2%, respectively (*P* > 0.05), and the DCR was 92.2% and 86.4%, respectively (*P* > 0.05), indicating no significant difference in short-term efficacy between the two treatment modalities.(5)Survival status: The PFS in the proton group and X-ray group was 31.6 ± 3.5 months (95% CI: 24.7 ~ 38.5) and 24.9 ± 1.55 months (95% CI: 21.9 ~ 27.9), respectively (*P* > 0.05), while the OS was 51.6 ± 4.62 months (95% CI: 42.5 ~ 60.7) and 33.1 ± 1.99 months (95% CI: 29.2 ~ 37.1), respectively (*P* < 0.05).According to the annual-specific analysis, the PFS rates for the first to third years in both groups were as follows: 100%, 56.1% and 32.5% for the proton group vs. 100%, 54.3% and 26.3% for the X-ray group. No statistical differences were observed at each time point (*P* > 0.05).The OS rates for the first to third years in both groups were as follows: 100%, 88.2%, 76.4% for the proton group vs. 100%, 91.4%, 46.3% for the X-ray group. There was no significant difference in the first to second years (*P* > 0.05), but the third year showed a significant difference (*P* < 0.05). Survival curve graphs also depicted a similar trend.

**Conclusion:**

There were no significant statistical differences observed between the two groups in terms of PFS and OS within the first two years. However, the proton group demonstrated a clear advantage over the X-ray group in terms of adverse reactions and OS in the third year. This suggests a more suitable treatment modality and clinical evidence for populations with frail health, compromised cardio-pulmonary function, post-COVID-19 sequelae, and underlying comorbidities.

## Introduction

Lung cancer is a significant cause of cancer-related deaths in humans, with non-small cell lung cancer (NSCLC) accounting for 85% of all lung cancer cases. Among NSCLC patients, approximately 80% are diagnosed at advanced stages (III/IV) or with unresectable disease. Histologically, NSCLC includes adenocarcinoma, squamous cell carcinoma, large cell carcinoma, and sarcomatoid carcinoma, among others [1]. According to the GlobalData report on NSCLC, in 2015, eight major countries worldwide accounted for over 90% of the NSCLC market, with China representing approximately 8% of that market. It is projected that by 2025, the market in Asian countries like China and Japan will continue to expand, comprising approximately 38.4% of the global NSCLC market, posing significant challenges for regional healthcare [2].

The PACIFIC study/real-world research has initiated and established the role of immune maintenance therapy following concurrent chemoradiotherapy in driver gene-negative locally advanced non-small cell lung cancer (NSCLC), significantly enhancing anti-tumor activity and survival benefits. This treatment approach has now received category 1 recommendations in international and regional organizations such as the National Comprehensive Cancer Network (NCCN), the European Society for Medical Oncology (ESMO), the Japan Society of Clinical Oncology (JSCO), the Chinese Society of Clinical Oncology (CSCO) and is widely recognized and utilized [3]. However, not all patients are suitable for this treatment approach. For instance, elderly patients, those with a general condition of ECOG PS > 2, individuals with underlying cardio-pulmonary conditions, post-COVID-19 sequelae, negative immune therapy-related gene mutations, and other factors, may not be candidates for standard concurrent chemoradiotherapy and immune maintenance therapy.At present, major guidelines do not provide further specific recommendations or elaborations for this subset of patients. It is generally recommended to optimize radiation therapy techniques to enhance clinical benefits, but there are no more specific research findings. Proton therapy(PT) is one of the most advanced and precise radiation therapy modalities currently available, offering superior physical characteristics compared to traditional X-ray treatment. It enables precise targeting and eradication of tumor lesions while minimizing radiation exposure to surrounding normal tissues, including critical organs such as the heart, lungs, spinal cord and esophagus.Clinical treatment data indicates that PT achieves an efficacy rate of over 95%, being evaluated by both the high-energy physics and medical communities as the most effective and least side-effect-prone treatment method [4]. In addition to its local effects, the robust radiation of protons, compared to traditional X-rays, is more capable of eliciting the body’s immune response. By activating CD8 + T lymphocytes mediated by dendritic cells, it can achieve immune sensitization effects [5]. The PT approach holds promise for providing new treatment opportunities to driver gene-negative, locally advanced non-small cell lung cancer (NSCLC) patients who are unable to receive or tolerate conventional chemoradiotherapy and immune maintenance therapy. It is anticipated to improve survival benefits. In conclusion, this study addresses real-world clinical challenges and, through the observation and analysis of different treatment modalities (chemoradiotherapy + immune maintenance with X-rays vs. concurrent chemoradiotherapy with protons) in the Asian population, provides clinical evidence for exploring new treatment modalities for driver gene-negative, locally advanced NSCLC.

## Materials and methods

### Patients

This study conducted a retrospective analysis of Asian patients who underwent X-ray or proton radiation therapy at two regional medical centers from September 2010 to September 2022.(1)Department of Radiotherapy, The Affiliated Hospital of Qingdao University(China). Inclusion Criteria: (a) Histopathologically confirmed non-small cell lung cancer with negative driver genes; (b) Clinical stage of locally advanced; (c) ECOG PS < 2; (d) Curative radiation therapy using X-ray-based IMRT techniques; (e) No history of severe cardio-pulmonary diseases or COVID-19; (f) No negative gene mutations associated with immunotherapy checkpoint inhibitors; (g) Normal blood routine and liver and kidney function.Exclusion Criteria: (a) Presence of distant metastases; (b) Incomplete data recording, insufficient information; (c) Patient refusal or treatment interruption.(2)Department of Radiation Oncology, University of Tsukuba Hospital (Japan). Inclusion Criteria: (a) Histopathologically confirmed non-small cell lung cancer with negative driver genes; (b) Clinical stage of locally advanced; (c) ECOG PS < 2; (d) Curative radiation therapy using proton-based techniques; (e) Allowance for cardiovascular and pulmonary diseases with specific requirements: NYHA heart function grade < 3, GOLD pulmonary function grade < 3; (f) Allowance for a history of severe COVID-19, provided there has been recovery; (g) Normal blood routine and liver and kidney function.Exclusion Criteria: Same as the X-ray group.

### Anti-tumor treatment regimen

#### Local treatment (radiation therapy)

a)X-ray group:Dosage 60–66 Gy/30–33 fractions/6–7 weeks (single dose 2 Gy), once daily radiation therapy, from Monday to Friday, a total of 5 times per week, using IMRT radiation therapy technique, with 5 fields or more, and IGRT performed every other day. Target area definition follows the ICRU-62 document, and organ-at-risk constraints refer to QUANTEC’s TD5/5, including the spinal cord, lungs, esophagus, heart, etc.

b)Proton group:Dosage 66 GyE/33 fractions/7 weeks (single dose 2 GyE), once daily radiation therapy, from Monday to Friday, a total of 5 times per week, with fixed-angle irradiation using 2 fields. All other parameters are the same as the X-ray group.

#### Systemic treatment (chemotherapy and immune checkpoint inhibitors)

a)X-ray group:Synchronous Chemotherapy Regimen: Paclitaxel Albumin-bound 175 mg/m2 IV on Day 1 + Cisplatin 75 mg/m2 IV on Day 1, every 3 weeks, for 4–6 cycles.Immune Checkpoint Inhibitor Maintenance Treatment Regimen: PD-1 inhibitor 200 mg IV on Day 1, every 3 weeks, for maintenance therapy over 1 year.

b)Proton group:Synchronous Chemotherapy Regimen: Paclitaxel Albumin-bound 175 mg/m2 IV on Day 1 + Cisplatin 75 mg/m2 IV on Day 1, every 3 weeks, for 4–6 cycles.

### Clinical efficacy and toxicity assessment

(1)Clinical efficacy assessment followed the RECIST v1.1 evaluation criteria [6], with monthly evaluations. Categories included: Complete Response (CR), Partial Response (PR), Stable Disease (SD) and Progressive Disease (PD). Relative Response Rate (RR) = CR + PR and Disease Control Rate (DCR) = CR + PR + SD.

(2)Toxicity assessment primarily followed the CTCAE v3.0 evaluation criteria [7], with weekly evaluations. The assessment is categorized from Grade 0 to Grade 5, with a focus on adverse reactions of Grade 2 or higher in this study.

### Follow-up and statistical analysis

(1)Follow-up:At 1 month after treatment completion, every 3 months within the first 2 years, every 6 months in the 3rd to 5th year, and annually after 5 years. Follow-up includes monitoring patient symptoms, physical examination, blood routine, biochemistry, tumor markers, pulmonary fibrosis markers, and imaging examinations.

(2)Statistical Analysis:All data were statistically analyzed using GraphPad Prism v9.0. The T-test was employed with *P* < 0.05 considered statistically significant for differences, and Kaplan-Meier analysis was used to assess patient survival.

## Results

### Patient clinical characteristics

A total of 62 driver gene-negative, inoperable stage IIIB (UICC staging, 8th edition) NSCLC patients who underwent treatment at Department of Radiotherapy, The Affiliated Hospital of Qingdao University(China), and Department of Radiation Oncology, University of Tsukuba Hospital (Japan) from September 2010 to September 2022 were included in the study. Of these, 37 patients were in the X-ray treatment group, and 25 patients were in the PT group. Specific clinical data can be found in Table 1.

**Table 1:** Patient Clinical Data

Clinical characteristics	*n*	Percentage(%)
Gender		
Male	39	62.9
Female	23	37.1
Age(yr)		
Median(range)	76(41~90)	
≦ 60	14	22.6
>60	48	77.4
History of smoking		
Yes	42	67.7
No	20	32.3
T stage		
T1	7	11.3
T2	17	27.4
T3	16	25.8
T4	22	35.5
N stage		
N1	1	1.6
N2	35	56.5
N3	26	41.9
Pathologic pattern		
Adenocarcinoma	29	46.8
Squamous cell carcinoma	27	43.5
Others	6	9.7
Treatment		
RT(X-ray)+ Chemo → ICI	37	59.7
RT(Proton) + Chemo	25	40.3

### Radiation therapy plan and parameters

**Fig. 1:**
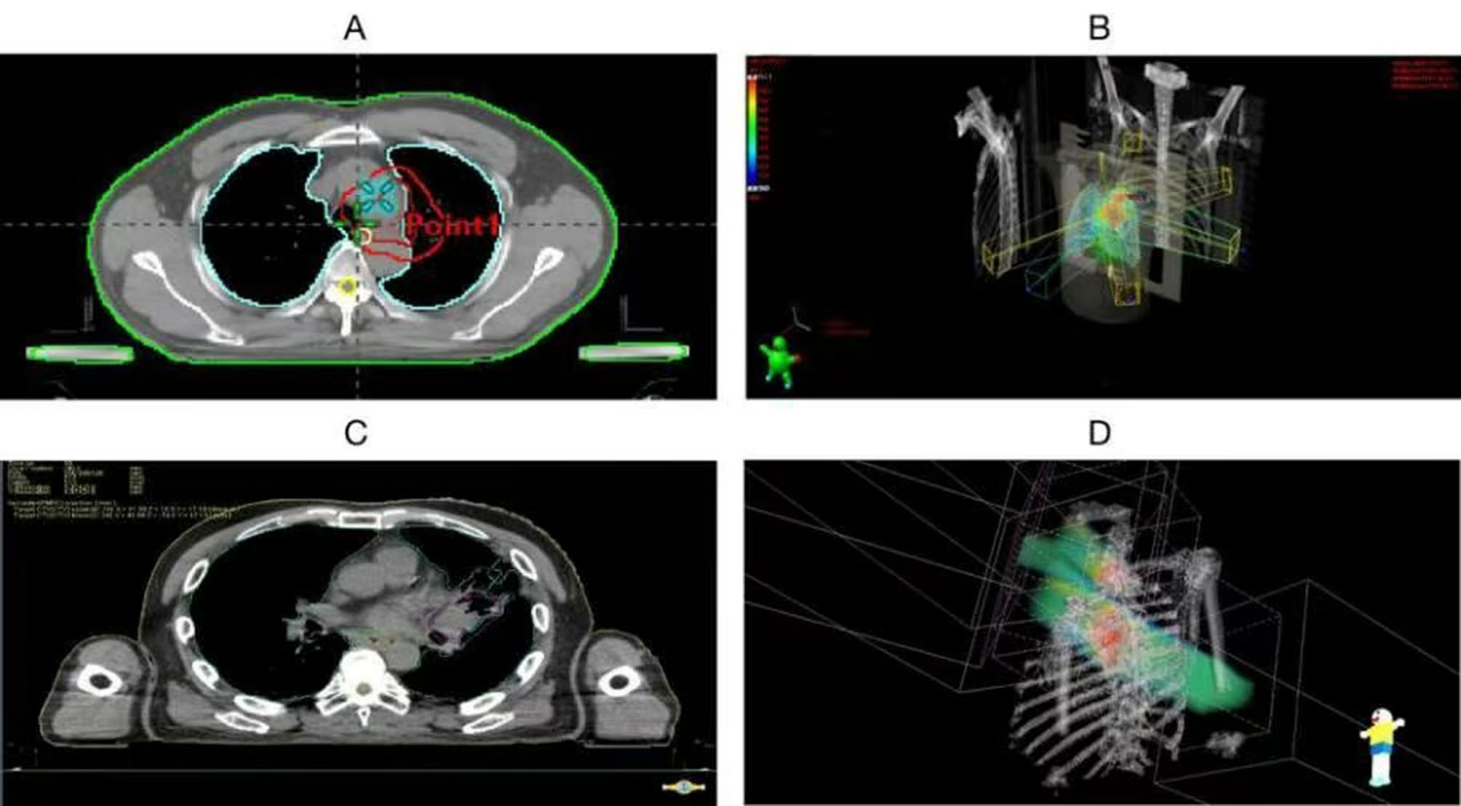
Radiotherapy Target Area and Field. Note: **A** and **B** represent the target area and field distribution for intensity-modulated radiation therapy (IMRT) with X-rays, respectively; **C** and **D** represent the target area and field distribution for fixed-angle proton therapy, respectively

**Fig. 2:**
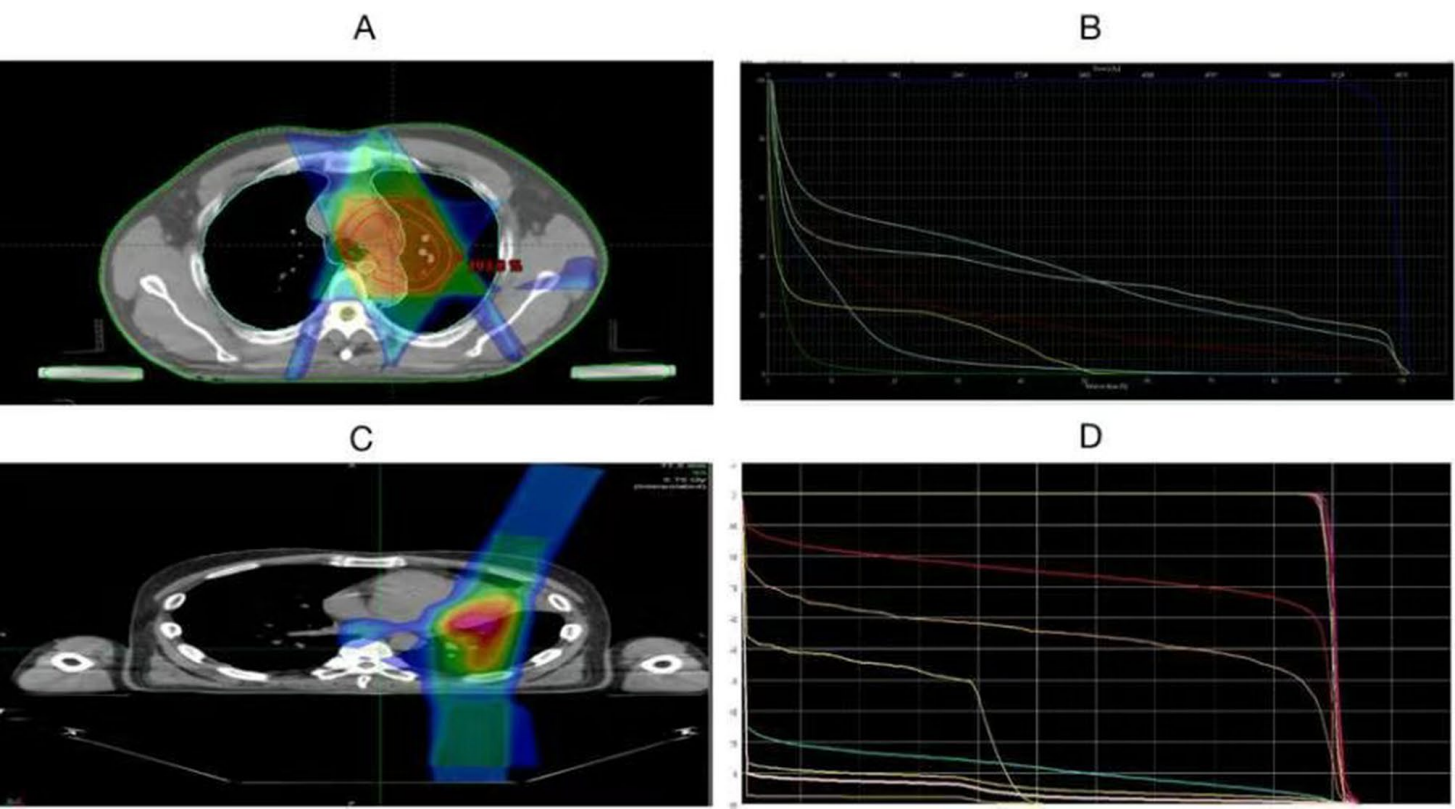
Dose Distribution and DVH. Note: **A** and **B** represent the dose distribution and DVH (Dose-Volume Histogram) for intensity-modulated radiation therapy (IMRT) with X-rays, respectively; **C** and **D** represent the dose distribution and DVH for fixed-angle proton therapy, respectively

The radiation target areas and doses are as shown in Fig. 1; Table 2, respectively. The organs at risk and their respective radiation doses are presented in Fig. 2; Table 3.

**Table 2:** Radiation Therapy Doses and Delivery Methods

Radiation Type	Average Age	Fractionation Scheme	Total Dose	Single Dose	Cardiopulmonary Diseases
X-ray	65	Convention fraction	60~66Gy	2 Gy	64%
Proton	71	Convention fraction	66 Gy	2 Gy	16%

**Table 3:** Organs at Risk and Doses Received(Mean ± SE)

Radiation Type	Lung			Spinal cord	Heart		Esophagus	
	V5 (%)	V20 (%)	MLD (Gy)	Dmax (Gy)	V40 (%)	Dmax(Gy)	Dmax(Gy)	Dmean(Gy)
X-ray	56.72 ± 1.89	23.95 ± 0.12	14.01 ± 0.20	39.75 ± 0.67	16.75 ± 0.42	68.49 ± 0.34	67.06 ± 0.28	31.59 ± 0.21
Proton *P* t df	26.22 ± 1.41 *** 11.83 60	19.79 ± 0.94 *** 5.31 60	10.98 ± 0.39 *** 7.54 60	40.57 ± 0.55 0.38 0.88 60	2.79 ± 0.11 *** 26.84 60	54.36 ± 0.21 *** 31.47 60	67.04 ± 0.17 0.96 0.05 60	32.67 ± 1.95 0.51 0.67 60

Note: Statistical results are presented as mean ± standard error, with P < 0.05 considered statistically significant(***: *P*<0.001, **:*P*<0.01, *:*P*<0.05)

### Toxicity and evaluation

**Fig. 3:**
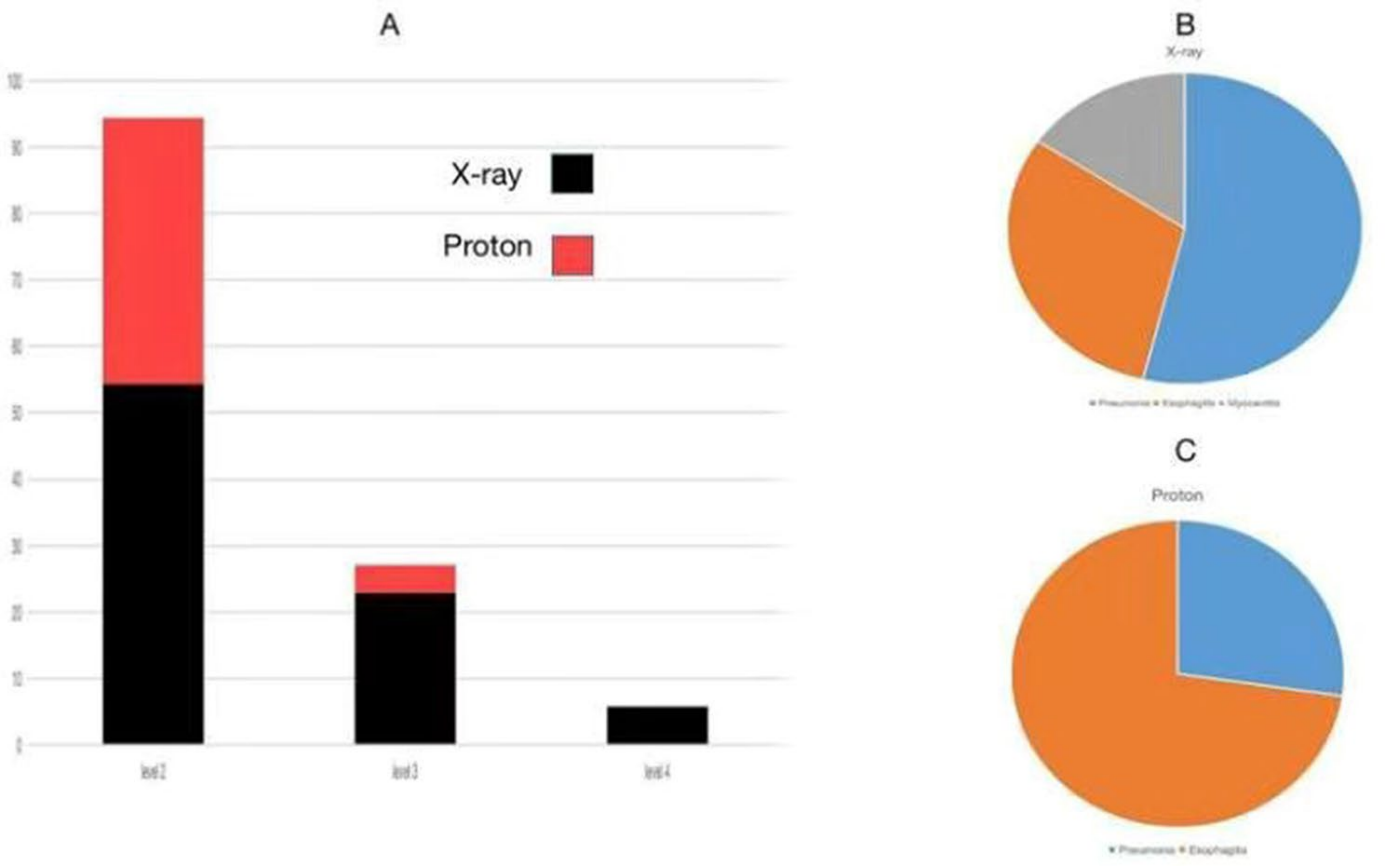
Adverse Event Severity and Types. Note: **A** represents the severity and proportions of adverse events induced by X-rays (black) and proton therapy (red); **B** and **C** depict the types and proportions of adverse events induced by X-rays (Pneumonia: blue, Esophagitis: orange, Myocarditis: gray) and proton therapy

(1) Regarding the severity of adverse events, the proportions of grade 2, grade 3, and grade 4 adverse events induced by X-ray and PT were 54.3%, 22.9% and 5.7% versus 40.1%, 4.2% and 0%, respectively (Fig. 3A), with a significant difference between the two groups (*P* < 0.05).

(2) In terms of the types of adverse events, X-ray therapy primarily induced pneumonia, esophagitis, and myocarditis, with proportions of 53.8%, 30.7% and 15.5%, respectively (Fig. 3B); while PT mainly led to esophagitis and pneumonia, with proportions of 72.7% and 27.3%, respectively, without occurrences of myocarditis (Fig. 3C). None of the cases experienced radiation-induced myelopathy or esophagotracheal fistula.

### Short-term efficacy assessment

X-ray treatment group: 2 cases achieved Complete Response (CR), 24 cases had Partial Response (PR), 6 cases exhibited Stable Disease (SD), and 5 cases showed Progressive Disease (PD), resulting in a Relative Response Rate (RR) of 70.2% and a Disease Control Rate (DCR) of 86.4%.PT group: 4 cases achieved CR, 13 cases had PR, 6 cases exhibited SD, and 2 cases showed PD, resulting in a RR of 68.1% and a DCR of 92.2%.Comparison of short-term efficacy between the two groups revealed no statistically significant difference (*P* > 0.05).

### Survival analysis

The median follow-up time was 30.9 months (range: 7.2 months to 108.2 months), during which 14 patients died. The causes of death were as follows: disease progression in 9 patients, radiation-induced pneumonia in 3 patients, and cardiovascular events in 2 patients, with the latter two occurring exclusively in the X-ray treatment group. The specific survival outcomes for both groups are as follows.

**Fig. 4:**
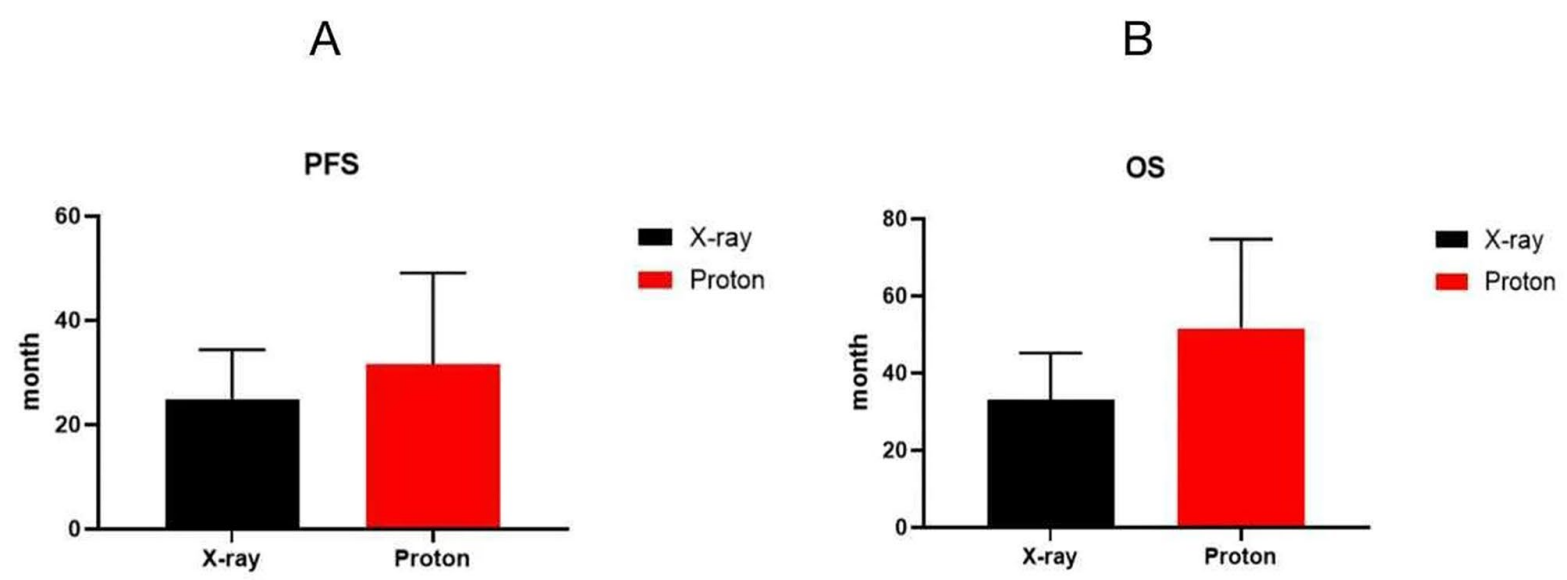
PSF and OS for Two Groups. Note: The vertical axis represents time (months), the horizontal axis represents groups, and **A** and **B** represent PFS and OS, respectively. The black bars represent the X-ray group, and the red bars represent the Proton group

**Fig. 5:**
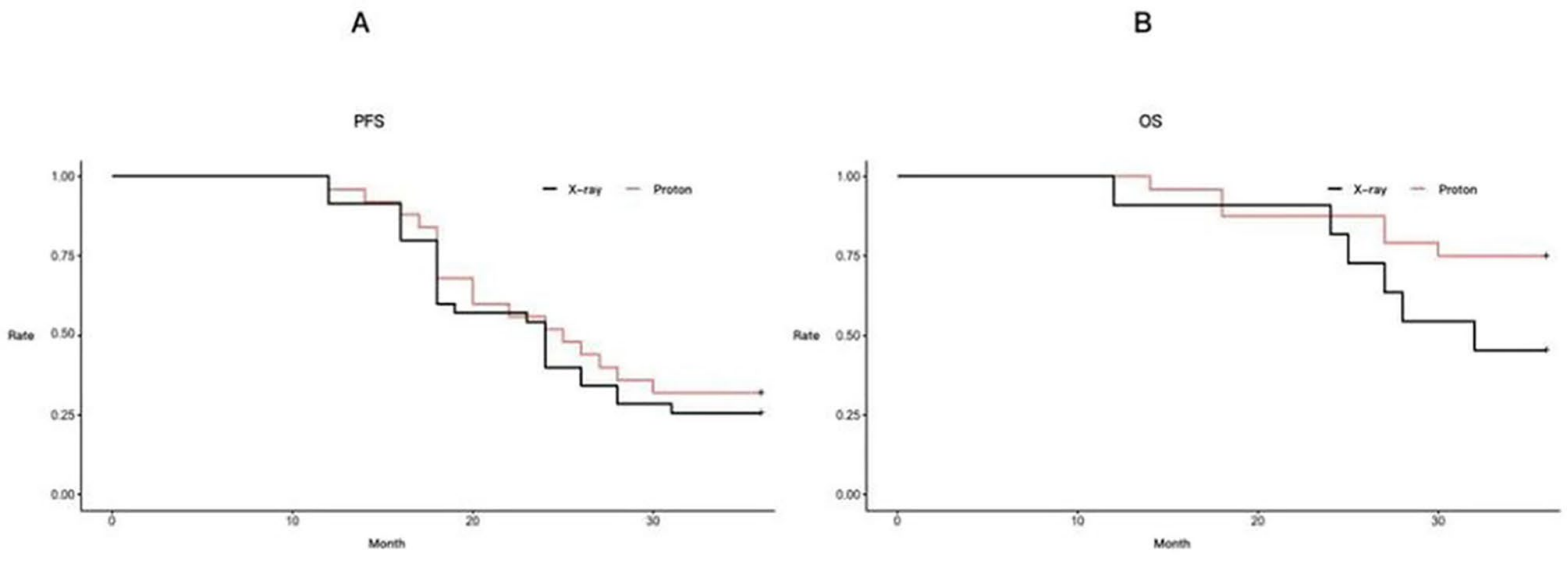
PSF Rate and OS Rate for Two Groups. Note: The vertical axis represents rates (%), the horizontal axis represents time (months), and A and B represent PFS and OS, respectively. The black lines represent the X-ray group, and the red lines represent the Proton group

(1) PFS: The PFS for the X-ray treatment group was 24.9 ± 1.55 months (95% CI: 21.9 ~ 27.9), while it was 31.6 ± 3.5 months (95% CI: 24.7 ~ 38.5) for the PT group, with no statistically significant difference between them (*P* > 0.05, t = 1.891, df = 58). (Fig. 4A)

(2) OS: The OS for the X-ray treatment group was 33.1 ± 1.99 months (95% CI: 29.2 ~ 37.1), while it was 51.6 ± 4.62 months (95% CI: 42.5 ~ 60.7) for the PT group, showing a statistically significant difference between the two (*P* < 0.05, t = 2.491, df = 33). (Fig. 4B)

(3) PFS rates: The PFS rates for the X-ray treatment group at the first, second, and third years were 100%, 54.3% and 26.3%, respectively. For the PT group, the corresponding rates were 100%, 56.1% and 32.5%. There was no statistically significant difference between the two groups at any of these time points (*P* > 0.05). (Fig. 5A)

(4) OS rates: The OS rates for the X-ray treatment group at the first, second, and third years were 100%, 91.4% and 46.3%, respectively. In contrast, for the PT group, the corresponding rates were 100%, 88.2% and 76.4%. There was no statistically significant difference between the two groups in the first two years (*P* > 0.05), but a statistically significant difference emerged in the third year (*P* < 0.05). (Fig. 5B)

## Discussion

According to Datamonitor Health’s predictions, China and Japan are expected to be the major regions for the future incidence of non-small cell lung cancer. Moreover, a significant portion of these cases will be unresectable locally advanced patients, posing increasing challenges to the healthcare environment. [8, 9]. In recent years, the emergence of targeted therapies and immune checkpoint inhibitors has improved clinical outcomes for a subset of patients with genetic mutations and high expression. However, these treatments are not applicable to the majority of patients [10, 11]. Additionally, drug resistance and relapse remain significant drawbacks and when considering the large number of affected individuals, the overall treatment effectiveness is far from ideal [12]. Over the past decade, several international clinical trials have been conducted for locally advanced non-small cell lung cancer patients who are negative for driver gene mutations and exhibit low expression levels. Unfortunately, the outcomes of these trials have all ended in failure. It is worth noting that previous studies on stage III synchronous chemoradiotherapy or the PACIFIC series have primarily focused on individuals with good physical condition (ECOG < 2), while excluding special populations such as the elderly, those with frail health, individuals with underlying cardiopulmonary conditions, those with post-COVID-19 sequelae and others from clinical research.In this population, survival benefits are often poorer. Sabine et al. concluded, through the analysis of 161 inoperable non-small cell lung cancer patients receiving synchronous chemoradiotherapy, that besides T stage, cardiopulmonary function variables also affect the post-chemoradiotherapy survival rate of non-small cell lung cancer patients [13]. John et al. conducted a multicenter meta-analysis and observed that 20% of patients receiving synchronous chemoradiotherapy experienced grade 3 or higher adverse events, with only 45% of patients able to receive standard synchronous chemoradiotherapy on schedule. These patients typically exhibit good physical condition, are under the age of 60 and have no underlying health conditions [14–16]. Jin et al. reported that in patients with locally advanced lung cancer who achieved a partial response (PR) to anti-tumor treatment, during the maintenance therapy phase with single-agent immune checkpoint inhibitors, some experienced an outbreak of fulminant acute myocarditis and eventually died due to the initiation of a cytokine storm within the body. Further analysis revealed that patients with interstitial lung disease and negative gene mutations had a higher likelihood of developing a systemic cytokine storm after receiving immune checkpoint inhibitors [17]. In summary, there is currently a lack of evidence-based medicine and treatment consensus for the specific population of patients with locally advanced non-small cell lung cancer mentioned above. Therefore, it is urgent to focus on this special population and explore better treatment strategies to improve clinical outcomes. Additionally, there is a need to enrich the evidence-based medical evidence for this special population and promptly share relevant databases.

**Fig. 6:**
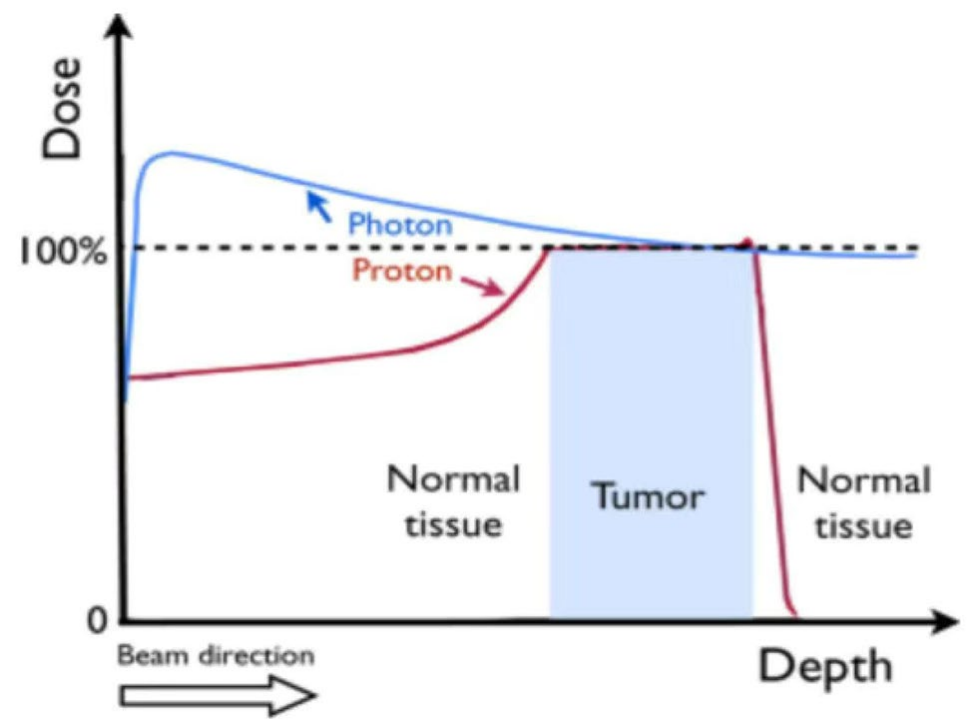
Schematic Dose-Depth Profiles for X-rays and Proton Beams. Note: Citing reference from [20]

The treatment effectiveness of cancer is closely related to advances in radiation therapy technology [18]. Protons, as particles, are different from photons such as X-rays and gamma rays; they continuously slow down as they change in depth. This process of dose deposition creates the characteristic depth-dose curve of proton beams, known as the “Bragg peak curve” as shown in Fig. 6 [19, 20]. Protons have excellent physical properties that allow for optimizing the dose distribution between tumors and surrounding normal tissues, as well as the DVH of organs at risk. This ensures treatment effectiveness while reducing adverse reactions. This has important clinical implications for the special high-risk population we mentioned earlier. It is also a significant reason for the substantial reduction in secondary primary tumors, which is beneficial for the trend of cancer becoming a chronic disease and the increasing incidence of cancer in younger populations [21, 22]. Interestingly, several studies have shown that PT can elicit a stronger immune response within the body compared to X-ray therapy, acting as a radiosensitizer. Randall et al. reported that PT induces DNA damage in tumor cells, leading to the release of cGAMP into the cytoplasm, which is subsequently released via the STING pathway to trigger IFN-1 production. This, in turn, is presented to CD8 + T cells by dendritic cells. Activated CTLs then attack tumor cells and tissues with the same antigen throughout the body, resulting in a widespread anti-tumor effect, known as the abscopal effect [23].

Based on the aforementioned research background and the physical-biological characteristics of PT, this study chose two Asian countries, Japan and China, with similar dietary, lifestyle, and geographic factors. These two countries, along with the United States, represent major growth markets for future non-small cell lung cancer. The results of this study are also of significant reference value. The University of Tsukuba Hospital in Japan has advanced expertise in the field of PT and has accumulated substantial experience [24]. Similarly, The Affiliated Hospital of Qingdao University in China is also a regional medical center with advanced radiation therapy equipment and capabilities, making it well-suited for the successful execution of this study. The study strictly adhered to the selection criteria for enrolled cases and divided them into two groups: one group receiving X-ray therapy, recommended for synchronous chemoradiotherapy and immune maintenance therapy according to NCCN guidelines and the other group receiving PT. The latter group received regular follow-up examinations due to subjective or objective reasons, with patients in this group having a higher average age and a higher incidence of comorbid heart and lung diseases compared to the former group.We first observed clinical efficacy and found that the RR and DCR of the X-ray group and the proton group were 70.2%, 86.4% vs. 68.1%, 92.2%, respectively. The short-term efficacy of patients in both groups was similar, with no statistically significant difference (*P* > 0.05). This is consistent with the results reported by Olsi et al. [25]. Longer-term follow-up results showed:(1)PFS: The X-ray treatment group had a median PFS of 24.9 ± 1.55 months (95% CI: 21.9 ~ 27.9), while the PT group had a median PFS of 31.6 ± 3.5 months (95% CI: 24.7 ~ 38.5), with no statistically significant difference between the two groups (*P* > 0.05, t = 1.891, df = 58) (Fig. 4A).(2)OS: The X-ray treatment group had a median OS of 33.1 ± 1.99 months (95% CI: 29.2 ~ 37.1), while the PT group had a median OS of 51.6 ± 4.62 months (95% CI: 42.5 ~ 60.7), with a statistically significant difference between the two groups (*P* < 0.05, t = 2.491, df = 33) (Fig. 4B).Sejpal et al. reported a retrospective comparative analysis of the experience at MD Anderson Cancer Center in stage III NSCLC patients, similarly finding no difference in median survival time between PT and X-ray synchronous chemoradiotherapy, with a *P*>0.05 (*P* = 0.1) [26]. Interestingly, although there were no differences in short-term efficacy and PFS between the two groups, there was a difference in OS. As shown in the survival curve in Fig. 5, with the passage of time, especially beyond the third year, the PT group showed a significant survival benefit over the X-ray group, and the difference appeared to be increasing. We consider that this long-term survival benefit may be related to the following two aspects. One is the significant reduction in long-term mortality due to cardiac and pulmonary toxicity by proton radiotherapy. The other is the unique dose-biological effect of PT, which can stimulate the immune response in the body and translate into better long-term survival, similar to the long tail effect of immunotherapy. The specific mechanisms require further basic research.

The above comparative results provide ample evidence of the advantages of PT. Next, let’s explore the specific differences between the two treatment modalities and the occurrence of adverse reactions. As shown in Fig. 1, the target area and irradiation range in the PT group are smaller than those in the X-ray group. This is related to the high physical characteristics and precision of PT. Typically, a patient’s PT plan is completed by 2 ~ 3 sub-plans, while X-ray therapy only has one plan. Therefore, PT can better protect the surrounding normal tissues. The dose distribution map and DVH in Fig. 2 confirm this result. We can see that the PT group has a more precise dose distribution and Bragg peak effect.Toshiki et al. compared the DVH parameters between PT and X-ray conformal radiotherapy (XCRT) in the treatment of locally advanced non-small cell lung cancer (NSCLC). The results showed that the average normal lung dose and V5 to V50 in PT were significantly lower than in XCRT [27]. The specific data from this study are shown in Table 3. First, the prescribed doses in both groups were within the allowed range according to RTOG’s TD5/5. Looking at specific areas, although there was no difference in the doses received by the spinal cord and esophagus between the two groups, in the lung (V5, V20, MLD) and heart (V40, Dmax), the PT group was significantly lower than the X-ray group, with statistical significance (*P* < 0.05).In other words, when achieving equivalent biological doses to the target lesion, the PT group can better protect the lungs and heart compared to the X-ray group. This result also explains the phenomenon in Table 2, where the X-ray group had a significantly higher incidence of combined heart and lung diseases after treatment compared to the PT group (64% vs. 16%). We further analyzed the occurrence of adverse events in all patients after treatment (Fig. 3). The incidence of grade 3 or higher adverse events in the X-ray and PT groups was 28.6% vs. 4.2%, showing a significant difference between the two groups. Upon further observation, it was found that the X-ray group mainly experienced pneumonia (53.8%), esophagitis (30.7%), and myocarditis (15.5%), while the PT group mainly experienced esophagitis (72.7%) and pneumonia (27.3%). In other words, the X-ray group exhibited higher cardiopulmonary toxicity, which is risky for individuals with impaired cardiopulmonary function.

## Conclusions

In conclusion, our research team, with extensive experience in frontline clinical work, embarked on this study driven by questions and interests, addressing practical challenges. Currently, apart from early-stage non-small cell lung cancer, locally advanced non-small cell lung cancer is not included in the indications for PT and is typically not covered by medical insurance, making it an uncommon choice. Therefore, there is a lack of relevant controlled study data for this population.Clinical commissioning of new indications is a hot topic in proton therapy, with different countries following different approaches.The model based for the Netherlands, well defined list of indications for the UK, with “grey areas” left to clinician evaluation on a patient basis.There are very limited data on the use of PT in NSCLC in adults, however, the use of PT in malignancies of the thorax has not demonstrated unexpected toxicities. In fact, the incidence of toxicities is reported to be comparable to X-Ray series in the biggest prospective registry published to date [28]. The capacity of PT and consequently its access is increasing worldwide, with potential new indications included in the commissioned list soon,not only primary disease, but reirradiation, oligometastatic diseases [29–32]. Even though the definitive answer on new indications for PT will come from randomised controlled trials, the historical difficulty of conducting RCT in proton therapy has hindered expansion of commissioning. Therefore, some countries have proposed evaluative commissioning strategies to expand the role of PBT, based on a strong clinical and physical rationale, corroborated by evidence of favourable toxicities profiles. In these contexts, prospective registries have become the mainstream, in order to collect real world PT patient data and fill in the gap in knowledge. The importance of granular and objective collection of toxicity data cannot be understated [33]. Through the observation and comparison of two groups from authoritative institutions, we have found that for inoperable locally advanced non-small cell lung cancer, the combination of synchronous radiochemotherapy (X-ray) followed by immunotherapy (as recommended by Category 1 guidelines) and PT with concurrent chemotherapy achieve similar short-term efficacy, with lower treatment toxicity in the latter case.This is highly valuable for the selection of treatment options for the special populations we have emphasized earlier (those with poor physical condition, advanced age, underlying cardiopulmonary diseases, severe post-COVID-19 sequelae, specific genetic mutations, etc.). Therefore, compared to palliative symptomatic treatment traditionally provided to this subset of individuals, the choice of a highly precise PT combined treatment model offers superior survival benefits. Additionally, in the future, further optimization of stratified treatment strategies based on enriched sample sizes and stratified analysis results is necessary to reflect the significance of individualized treatment and provide clinical evidence for evidence-based medicine databases. However, it should be noted that PT also has limitations such as high costs. With the increasing proliferation of PT technology and the miniaturization of equipment, these limitations are expected to be addressed in due course.

## Data Availability

All data generated or analysed during the current study are included in this published article.
